# Epigenetic Alterations in Oesophageal Cancer: Expression and Role of the Involved Enzymes

**DOI:** 10.3390/ijms21103522

**Published:** 2020-05-15

**Authors:** Nair Lopes, Margareta P. Correia, Rui Henrique, Carmen Jerónimo

**Affiliations:** 1 Cancer Biology and Epigenetics Group, Research Centre of Portuguese Oncology Institute of Porto (GEBC CI-IPOP), Rua Dr. António Bernardino de Almeida, 4200-072 Porto, Portugal; nair.ribeiro.lopes@ipoporto.min-saude.pt (N.L.); margareta.correia@ipoporto.min-saude.pt (M.P.C.); henrique@ipoporto.min-saude.pt (R.H.); 2 Department of Pathology, Portuguese Oncology Institute of Porto (IPO Porto), Rua Dr. António Bernardino de Almeida, 4200-072 Porto, Portugal; 3 Department of Pathology and Molecular Immunology, Institute of Biomedical Sciences Abel Salazar, University of Porto (ICBAS-UP), Rua de Jorge Viterbo Ferreira, 228, 4050-313 Porto, Portugal

**Keywords:** oesophageal cancer, epigenetics, methylation, histone modifications

## Abstract

Oesophageal cancer is a life-threatening disease, accounting for high mortality rates. The poor prognosis of this malignancy is mostly due to late diagnosis and lack of effective therapies for advanced disease. Epigenetic alterations may constitute novel and attractive therapeutic targets, owing to their ubiquity in cancer and their reversible nature. Herein, we offer an overview of the most important studies which compared differences in expression of enzymes that mediate epigenetic alterations between oesophageal cancer and normal mucosa, as well as *in vitro* data addressing the role of these genes/proteins in oesophageal cancer. Furthermore, The Cancer Genome Atlas database was interrogated for the correlation between expression of these epigenetic markers and standard clinicopathological features. We concluded that most epigenetic players studied thus far are overexpressed in tumours compared to normal tissue. Furthermore, functional assays suggest an oncogenic role for most of those enzymes, supporting their potential as therapeutic targets in oesophageal cancer.

## 1. Introduction

Oesophageal cancer is a life-threatening disease, with about 600,000 new cases annually and high mortality (500,000 deaths annually) worldwide [1] and it comprises two major histological types: squamous cell carcinoma (ESCC) and adenocarcinoma (EAC). These are quite different tumour entities, displaying distinct incidence rates, global distribution, and biological characteristics. ESCC are overall more incident than EAC and more frequently found in developing countries whereas, in contrast, EAC prevails in developed regions [2]. These differences are thought to reflect distinct habits/lifestyles and, perhaps, different genetic backgrounds of the affected populations. ESCC are also genetically and phenotypically closer to other squamous carcinomas of different organs, whereas EAC resemble gastric adenocarcinomas [3,4]. Unfortunately, the absence of symptoms and difficulties in screening mean that most oesophageal cancers are diagnosed at late stages [5]. Although this partially explains the poor prognosis and the low survival rates (5-year survival of about 15%–20%) [6,7], the lack of effective therapies, including targeted therapies for advanced disease, further contributes to the dismal outcome of oesophageal cancer. Indeed, at present, patients are offered only the classical therapeutic approach of surgery, chemotherapy (such as combinations of carboplatin and paclitaxel or cisplatin with fluorouracil), and radiotherapy, either alone or in combination within various regimens, depending on tumour stage [8,9,10,11,12,13,14,15]. Targeted therapies available for oesophageal cancer are scarce and include targeting of PD-1, HER2 and VEGF [16,17].

**Figure 1:**
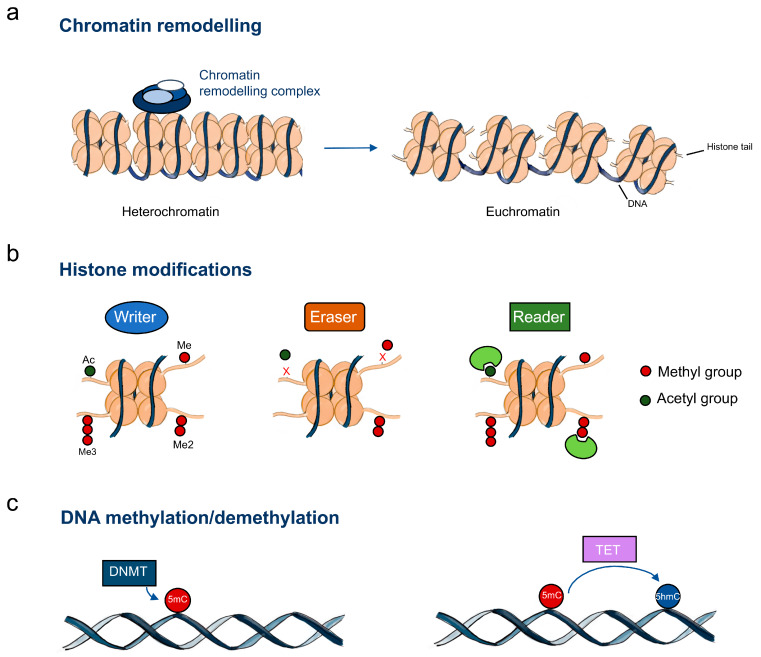
Epigenetic machinery in oesophageal cancer. (**a**) Chromatin-remodelling complexes catalyse chromatin changes including histone and DNA modifications; (**b**) Histone writers, erasers and readers; (**c**) DNA methylation regulators. Abbreviations: Me – methylation; Me2 – dimethylation; Me3 – trimethylation; Ac – acetylation; DNMT – DNA methyltransferases; TET – DNA demethylases; 5mC – 5-methylcytosine; 5hmC – 5-hydroxymethylcytosine.

Cancer has traditionally been regarded as a complex disease that arises as a multistep process through the accumulation of genetic alterations [18]. A new layer of complexity, however, has been added to our understanding of this illness and it is now widely accepted that epigenetic mechanisms contribute to the development of tumours, playing a role in the transformation of normal cells and controlling different events that constitute hallmarks of cancer [19]. Epigenetics consists of a series of heritable changes in chromatin structure that do not involve alterations of the DNA sequence itself [20]. It comprises three major mechanisms: DNA alterations, chromatin-remodelling processes (post-translational modifications of histones) and regulation by non-coding RNAs [21,22]. Among these, DNA methylation is possibly the most extensively studied epigenetic modification. Interestingly, in this regard, tumour cells constitute a paradox, since they are known to display global levels of hypomethylation, while at the same time exhibit promoter hypermethylation of specific genes [23]. In oesophageal cancer there is also evidence of deregulation of epigenetic mechanisms due to machinery abnormalities (Figure 1). For instance, it was observed that global hypomethylation was an early event in EAC development [24]. Importantly, epigenetic alterations have the advantage of being reversible, which makes them attractive therapeutic targets. One example is the possibility to reverse DNA hypomethylation through the use of SAM (S-adenosylmethionine), a donor of methyl groups to DNA. In various cancer models, treatment with SAM decreased proliferation, invasion and metastasis and promoted apoptosis of tumour cells [25]. Furthermore, combination of SAM with conventional chemotherapy drugs, such as doxorubicin and 5-fluorouracil, lead to synergistic anti-proliferative effects [26,27]. Interestingly, one study in breast cancer demonstrated the synergistic effect of the combination of SAM with decitabine, another epigenetic drug that inhibits DNA hypermethylation, hindering tumour growth and invasion [28]. In addition to SAM, inhibitors of DNA methyltransferases and histone deacetylases, as well as of other histone-modifying enzymes, are under investigation and have, in some cases, already reached clinical use, either alone or combined with conventional therapy, targeting various cancer models, including oesophageal cancer [29,30,31,32,33,34].

In this short review, a comprehensive analysis of epigenetic modifications in oesophageal cancer is presented, mainly focusing on the expression and role of the enzymes that mediate such alterations. Therefore, DNA and histone-modifying mechanisms will be discussed, but not regulation mediated by non-coding RNAs. Moreover, although histone-modifying enzymes can target non-histone proteins, the modification of such proteins will not be addressed by this review. First, studies that have used human tissue samples to evaluate differences in expression of these molecules between normal oesophageal epithelium and cancer are appraised. Subsequently, information from publicly available datasets is analysed to correlate the expression of epigenetic players with clinicopathological features, such as histological subtypes, tumour stage and the existence of lymph node metastasis. Finally, a short section is provided disclosing data from functional studies in which the role of epigenetic players was explored in the context of oesophageal cancer.

## 2. Epigenetic Players’ Deregulation in Oesophageal Cancer

This section discusses the published data on the expression levels of enzymes that mediate epigenetic modifications in human oesophageal tissue samples. The main goal was to look for differences in expression between normal and neoplastic tissue, and thus a PubMed search under “esophageal tumours AND (protein-coding epigenetic enzymes)” without time period limitations has been performed, where “(protein-coding epigenetic enzymes)” stands for the various family of epigenetic enzymes, for instance histone deacetylase/acetylase. Only articles written in English were examined and included. Information concerning mutations was included, when available, only for enzymes for which we could not find any data on expression. Unless otherwise stated, all comparisons and correlations cited are statistically significant. Additionally, the oesophageal cancer data from the publicly available The Cancer Genome Atlas (TCGA) database, as well as the normal data from TCGA and the GTEx (Genotype-Tissue Expression) Portal has been analysed, using the online resources cBioPortal for Cancer Genomics [35,36] and GEPIA [37]. Statistical analyses have been carried out using GraphPad Prism 7. In all analyses performed, *p* values < 0.05 were considered statistically significant. The figures refer to epigenetic enzymes that showed statistically significant results.

### 2.1. Histone Writers

#### 2.1.1. Histone Acetyltransferases

Histone acetyltransferases (HAT) catalyse the transfer of an acetyl group from acetyl-CoA to the lysine residues on the N-terminal tails of histones [38]. Their activity promotes chromatin unpacking, usually leading to transcription activation. KAT1/HAT1 (histone acetyltransferase 1) was found to be up-regulated in ESCC compared with adjacent tissues and normal oesophagus, and high expression of this enzyme was correlated with poorly differentiated tumours [39]. KAT3B/p300 was more expressed in ESCC in comparison with normal tissue and its expression directly associated with high histological grade, T category, N category, vascular thrombosis, and pathologic stage. Patients exhibiting low expression of KAT3B displayed better overall and disease-free survival in comparison to those with high KAT3B expression, and the expression of this enzyme was considered an independent prognostic factor for overall survival [40,41]. *KAT3B* transcript levels were associated with high histological grade, clinical stage, and lymph node involvement [41]. Interestingly, it has been reported that *KAT3B* promoter methylation levels were significantly higher in ESCC in comparison with non-tumour tissue. *KAT3B* promoter methylation was significantly more common in deeply invasive tumour areas than in less invasive tumour regions [42]. It was also demonstrated that in ESCC, *KAT3B* and *KAT3A*/*CREBBP* (CREB binding protein) harboured frequent inactivating mutations [43]. KAT13B/AIB1 (amplified in breast cancer 1) was overexpressed in ESCC in comparison with adjacent normal tissue [44] and its expression was positively associated with advanced clinical stage, distant lymph node metastasis and chemoradiotherapy resistance in ESCC [44,45,46,47]. Overexpression of KAT13B was linked with poor overall, disease-specific, and progression-free survival and was shown to be an independent predictor factor [45,47].

HAT enzymes promote oesophageal tumorigenesis and are associated with aggressive features of disease (Table 1).

**Figure 2:**
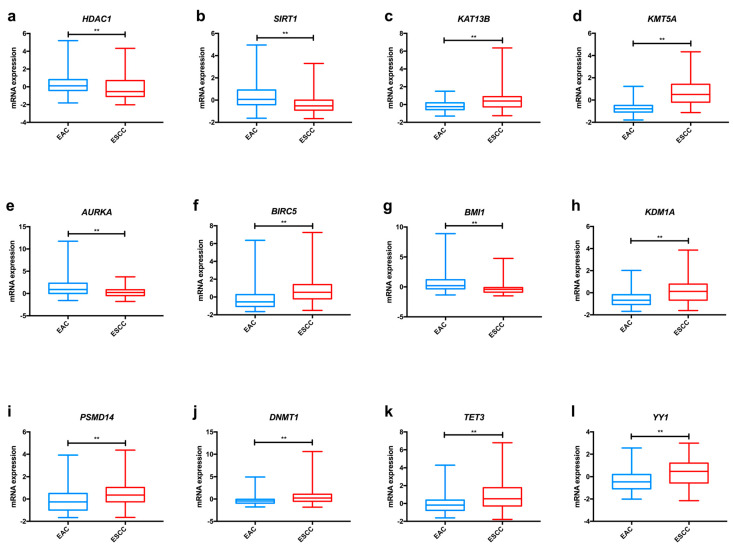
Examples of relevant modifications in the expression of enzymes involved in epigenetic alterations in oesophageal cancer, based on TCGA data analysed through the cBioPortal for Cancer Genomics resource. Differences in mRNA expression of *KAT13B* (**a**), *KMT5A* (**b**), *AURKA* (**c**), *AURKB* (**d**), *BMI1* (**e**), *HDAC1* (**f**), *SIRT1* (**g**), *KDM1A* (**h**), *PSMD14* (**i**), *DNMT1* (**j**), *TET3* (**k**) and *YY1* (**l**) between EAC and ESCC. Abbreviations: EAC – oesophageal adenocarcinoma; ESCC—oesophageal squamous cell carcinoma. ** *p* < 0.001, p value not adjusted for multiple comparisons.

**Figure 3:**
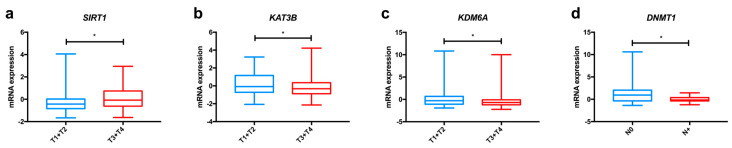
Relevant variations in the expression of enzymes involved in epigenetic alterations in oesophageal cancer, based on TCGA data analysed through the cBioPortal for Cancer Genomics resource. Differences in mRNA expression of *KAT3B* (**a**), *SIRT1* (**b**) and *KDM6A* (**c**) between early tumour stages (T1+T2) and advanced tumour stages (T3+T4); Differences in mRNA expression of *DNMT1* (**d**) between tumours with lymph node invasion and without lymph node invasion. Abbreviations: T1 to T4—tumour stages 1 to 4; N0—tumour sample without lymph node involvement; N+—tumour sample displaying lymph node metastasis; * *p* < 0.005.

In the TCGA dataset, we found significant differences among oesophageal cancer subtypes concerning *KAT1* and *KAT13B* expression (Figure 2a and Figure S1), with both enzymes displaying higher levels in ESCC in comparison to EAC. Furthermore, *KAT3B* expression levels are higher in early tumour stages (T1+T2) than in more advanced stages (T3+T4) of disease (Figure 3a).

#### 2.1.2. Histone Methyltransferases

Histone methyltransferases (HMT) are enzymes that catalyse the addition of one or more methyl groups into lysine (KMT) or arginine (PRMT) residues of histones. The effect on transcriptional activity depends on the histone and residue that is targeted, with H3K9me2/3, H3K27me2/3 and H4K20me3 associated with transcriptional repression, whereas H3K4me2/3 and H3K79me3 are linked with transcriptional activation [89,90]. *KMT1A*/*SUV39H1* (suppressor of variegation 3–9 homolog 1) trimethylates lysine 9 of histone H3 (H3K9me3). Its expression was up-regulated in ESCC compared to normal tissue and there was overexpression in late stages (III/IV) relative to early disease stages (I/II) [48]. KMT1C/G9a/EHMT2 (euchromatic histone lysine methyltransferase 2) that is involved in the methylation of several histone H3 residues, such as the mono- and dimethylation of lysine 9 (H3K9me1 and H3K9me2, respectively), the methylation of lysine 27 and lysine 56. Its expression seemed to increase from normal oesophageal epithelium to dysplasia and cancer, although these differences were not quantified, and it was directly associated with lymph node involvement, tumour dedifferentiation, late-stage, and depth of invasion. Moreover, KMT1C expression was considered an independent prognostic factor for overall survival [91]. KMT1D/EHMT1 that specifically mono- and dimethylates lysine 9 of histone H3 (H3K9me1 and H3K9me2, respectively), but also methylates lysine 27 of histone H3 was overexpressed in ESCC in comparison with normal epithelium and positively associated with high tumour grade, stage, depth of invasion and lymph node metastasis. At transcript level, *KMT1D* directly associated with high tumour grade, depth of invasion and lymph node involvement. Patients exhibiting high levels of KMT1D displayed poorer overall survival and the expression of this enzyme was considered an independent prognostic factor for ESCC [49]. Frequent truncating mutations were observed in the chromatin remodelling genes *KMT2C* and *KMT2D*, both responsible for the methylation of lysine 4 of histone H3 [43]. KMT3C/SMYD2 (SET and MYND domain containing 2) is an enzyme that methylates lysine 4 of histone H3 and dimethylates lysine 36 of histone H3 (H3K36me2). A positive correlation was observed between KMT3C expression and depth of tumour invasion, venous invasion, recurrence and worse overall survival, being KMT3C expression an independent prognostic factor [50]. KMT3E/SMYD3 is involved in the methylation of lysine 4 of histone H3, as well as the methylation of lysine 5 of histone H4. Its expression levels were higher in ESCC compared to matching non-cancerous tissue and were associated with lymph node metastasis [51,52]. Patients with low KMT3E expression displayed better overall survival, and it was considered an independent prognostic factor [52]. Regarding KMT5A (monomethylates lysine 20 of histone H4–H4K20me1), low protein expression associated with better prognosis [53], whereas *KMT6A/EZH2* (enhancer of zeste homolog 2, involved in the methylation of lysine 9 of histone H3, as well as in the mono-, di- and trimethylation of lysine 27 of histone H3–H3K27me1, H3K27me2 and H3K27me3, respectively) expression levels were up-regulated in tumour tissue comparatively to paired normal epithelium, positively associating with late tumour stage and lymph node metastasis [48,92]. There were also significantly higher KMT6A protein levels in ESCC than in adjacent normal tissue [48,54,55], associating with tumour location, high grade, larger size, greater depth of invasion, lymph node involvement, chemotherapy response and the presence of distant metastases [54,55,56]. Patients depicting KMT6A positivity had worse overall survival and patients displaying aberrant expression had poor disease-free, disease-specific, and progression-free survival [54,55,56]. Moreover, KMT6A expression was considered an independent prognostic factor for overall survival in ESCC [55]. Using a publicly available dataset (GSE20347), Koumangoye and collaborators [93] verified that *KMT6A* was overexpressed in ESCC compared to normal oesophageal tissue. Furthermore, using another dataset (GSE47404) the same authors found that *KMT6A* was significantly up-regulated in patients with metastatic disease [93].

In parallel with the HAT family of proteins, HMT proteins behave as promoters of oesophageal tumour development (Table 1). In the TCGA dataset, we found that ESCC exhibit significantly higher *KMT3C*, *KMT3E* and *KMT5A* expression levels in comparison with EAC (Figure 2b and Figure S1).

#### 2.1.3. Kinases

Kinases are enzymes that mediate the transfer of phosphate groups to specific substrates of target proteins. The effects of histone phosphorylation on gene regulation are rather disparate, as the same kinase can act on various residues, leading to different results. For instance, Aurora A is known to phosphorylate threonine 118 of histone H3, causing chromatin loosening; at the same time, phosphorylation of serine 10 of histone H3 by Aurora A decreases DNA accessibility [94,95,96]. Furthermore, the same histone residue can be targeted by more than one kinase with opposite effects in terms of transcriptional activity, adding a new layer of complexity to this type of regulation [97]. Aurora A was reported to be up-regulated in tumour tissue compared with normal adjacent mucosa [57,58,59] and its expression was higher in less differentiated tumours [57]. *AURKA* (Aurora kinase A)/Aurora A expression directly correlated with histological grade, lymph node invasion and TNM stage [58,59,60]. Chemoradiation therapy was shown to be effective in terms of clinical and histological effect in Aurora A-positive patients [98]. Regarding survival, poor overall and disease-free survival were observed for patients with Aurora A expression and it was also considered an independent prognostic factor [60]. Similar to Aurora A, Aurora B was up-regulated in tumour tissue compared with normal adjacent mucosa and its expression correlated with high histological grade, TNM stage and lymph node status [59,62]. Patients displaying high levels of Aurora B expression had poorer overall survival [62]. PKC (protein kinase C) expression was higher in tumour tissue than in the normal oesophageal mucosa [63]. *PRKCI* (protein kinase C iota) mRNA and protein expression levels were up-regulated in ESCC compared with normal tissue and the protein expression levels associated with larger tumour size, high grade and invasion [64]. High PKCι levels were correlated with poor overall survival and by multivariable analysis it was considered an independent prognostic factor [64]. PAK1 (p21 [RAC1] activated kinase 1) was up-regulated in primary oesophageal small cell carcinomas compared with adjacent normal tissue and positively associated with tumour location and lymph node metastasis. Patients exhibiting higher levels of PAK1 had poor prognosis [66]. Using TCGA data, Zhu and colleagues [67] demonstrated that *PAK2* was up-regulated in tumour tissue in comparison with normal samples and this result has been confirmed by protein expression in paired tumour/normal tissue samples.

Overall, kinase proteins associate with aggressive traits of oesophageal carcinomas (Table 1). In the TCGA dataset, *AURKA* was significantly more expressed in tumour tissue in comparison with normal oesophageal epithelium (Figure S3). *AURKB*, *PAK2* and *AMPK* expression levels were superior in ESCC compared with EAC, whereas the opposite was demonstrated for *PRKCA* and *AURKA* levels (Figure 2c,d and Figure S1).

#### 2.1.4. Ubiquitin-Activating/-Conjugating/-Ligation Enzymes

Ubiquitin-activating enzymes, ubiquitin-conjugating enzymes and ubiquitin ligases are enzymes that are involved in the activation, conjugation, and ligation steps of ubiquitination, respectively. This process results in the inclusion of an ubiquitin residue in a substrate protein. This type of histone modification occurs mostly on histones H2A and H2B [99]. Similar to phosphorylation, histone ubiquitination can cause both gene activation and transcriptional repression, depending on the targeted histone. H2A ubiquitination is mostly involved in gene silencing, whereas H2B modification often associates with transcriptional activation, although it also participates in transcriptional repression [100,101,102]. In ESCC, it was demonstrated that Smurf2 (SMAD specific E3 ubiquitin protein ligase 2) expression significantly and positively correlated with depth of invasion and lymph node involvement. Moreover, patients with Smurf2-positive tumours endured worse prognosis [68]. BMI1 (Bmi1 polycomb ring finger) was up-regulated in ESCC in comparison with adjacent normal oesophagus [54,69]. However, data on BMI1 associations with clinicopathological parameters were discrepant: some authors found that it inversely associated with lymph node invasion and venous involvement [54], whereas others reported direct associations with larger tumour size, late stages and lymph node invasion [69]. Nevertheless, the reason for this inconsistency is unknown [54]. Patients with high BMI1 expression exhibited shorter overall survival [69]. FBXO31 (F-box protein 31) expression in oesophageal tumour tissue displayed a significantly higher score than in paired normal mucosa [70], and it was associated with low histological grade but high clinical stage. Patients displaying high FBXO31 expression showed poorer prognosis and high FBXO31 expression was considered an independent prognostic factor [70,71]. Patients presenting low FBXW7 (F-box and WD repeat domain containing 7) levels showed worse overall survival [72]. Furthermore, *UBE2C* (ubiquitin-conjugating enzyme E2C) mRNA was overexpressed in EAC compared to Barrett’s metaplasia (although the authors did not quantify this difference) and protein immunostaining was observed in tumour tissue, whereas Barrett’s metaplasia samples were negative [73]. Likewise, there was overexpression of *UBE2C* in ESCC compared with non-malignant adjacent tissue [74,75] and both displayed significantly higher *UBE2C* expression levels compared to tissue from healthy donors. Interestingly, it was demonstrated that *UBE2C* expression discriminated tumour tissue from normal epithelium and tumour-surrounding tissue with high sensitivity and specificity [74]. UBE2C protein expression was positively correlated with lymphatic vessel involvement, lymph node metastasis, advanced clinical stage, N stage and metastasis status [73,76]. Moreover, patients with high UBE2C expression showed poorer relapse-free survival and it was considered an independent prognostic factor [76]. Furthermore, high SKP2 (S-phase kinase associated protein 2) levels were correlated with advanced tumour stage and lymph node invasion [103] and among patients that received chemotherapy and radiation therapy, those with high SKP2 expression levels had poorer overall survival [103]. RNF113A (ring finger protein 113A) was up-regulated in ESCC compared with the normal counterpart and it associated with poorly differentiated and late-stage tumours [77]. Accordingly, patients with low RNF113A expression displayed the best prognosis and RNF113A expression was an independent prognostic factor for overall survival in ESCC [77]. STUB1/CHIP (STIP1 homology and U-box containing protein 1/C-terminal Hsp-interacting protein) levels were higher in lymph nodes than in paired primary tumours. High CHIP expression in the lymph nodes predicted worse disease-specific survival and was considered an independent prognostic factor [104].

Again, as previously mentioned for other proteins, ubiquitination enzymes are important players in the acquisition and maintenance of the malignant phenotype in oesophageal cancer (Table 1). TCGA dataset analysis disclosed increased *BMI1* and *RNF113A* expression levels in EAC comparatively to ESCC (Figure 2e and Figure S1). Conversely, CHIP1/*STUB1* was up-regulated in ESCC compared with EAC (Figure S1). Furthermore, we found that patients with low *RNF113A* expression levels presented better overall survival (Figure S2).

### 2.2. Histone Erasers

#### 2.2.1. Histone Deacetylases

The enzymes responsible for the removal of acetyl groups from lysine residues of histones are named histone deacetylases (HDAC). They contribute to reduce chromatin accessibility and are, therefore, considered transcription corepressors. It has been shown that *HDAC1* was more expressed in oesophageal cancerous tissues than matching normal epithelium [48,105]. Furthermore, *HDAC1* levels increased at advanced tumour stages (III/IV) in comparison with early stages (I/II) [48]. Concerning *HDAC2*, mRNA expression levels were significantly superior in ESCC than in the corresponding normal tissue [48] and the same was observed for protein expression [78,106]. Similar to *HDAC1*, *HDAC2* levels were also significantly increased in stages III/IV compared with stages I/II [48]. Furthermore, high HDAC2 levels correlated with lymph node invasion, less differentiated tumours and lymphatic vessel permeation [48,107]. In an EAC dataset (GSE13937), *HDAC3* was found increased in tumour samples compared with normal tissues [93]. In contrast to previous results, however, Ahrens and collaborators [108] observed lower HDAC1, HDAC2 and HDAC3 expression in EAC and ESCC, compared to normal epithelium, although they have not quantified this difference. *HDAC4* mRNA and protein were significantly overexpressed in ESCC in comparison with matching normal tissue. Furthermore, high HDAC4 expression associated with poor differentiation, late T stage, N stage and advanced TNM stage. Patients with high HDAC4 expression endured shorter overall and progression-free survival and HDAC4 expression was an independent prognostic factor for overall survival [109]. Finally, *HDAC5* expression levels were found higher in ESCC tissue than in the corresponding normal tissue [48], but no clinicopathological correlates were explored.

Considering the sirtuins family, *SIRT1* (sirtuin 1) transcript levels were higher in ESCC than in the corresponding normal mucosa [48,110], as well as in late tumour stages (III/IV) in comparison with early stages (I/II) [48]. Similarly, SIRT1 was also significantly overexpressed in ESCC comparatively to paired normal epithelium [110] and associated with late tumour stage, TNM stage, positive lymph node status and worse overall and disease-free survival, constituting an independent prognostic factor for ESCC [110,111,112]. Interestingly, serum SIRT3 levels were up-regulated in ESCC patients in comparison with healthy donors [113]. Moreover, low SIRT3 expression was associated with better overall and disease-free survival, and SIRT3 expression was considered an independent prognostic factor for overall survival in ESCC [114,115]. Conversely, there was an association between low *SIRT4* mRNA expression and poor prognosis, and negative SIRT4 protein levels correlated with worse overall and disease-free survival. Overall and distant relapse were significantly increased in SIRT4-negative tumours comparatively to SIRT4-positive tumours [116]. *SIRT6* levels were significantly up-regulated in ESCC in comparison with the corresponding normal tissue and protein overexpression correlated with TNM status, moderately/well differentiated tumours and histological phenotype [117].

Altogether, these studies indicate that overexpression of HDAC proteins (except for SIRT4) seems to be a meaningful event in oesophageal carcinogenesis, favouring tumour progression and associating with poor survival (Table 2). Analysing the TCGA dataset, we observed significant differences concerning the expression of HDACs in oesophageal cancer. *HDAC3* and *HDAC4* were more expressed in ESCC, whereas EAC displayed higher *HDAC1* and *HDAC6* levels (Figure 2f and Figure S1). Additionally, *SIRT1* expression levels were higher in EAC in comparison with ESCC, as depicted in Figure 2g, and this gene was less frequently expressed in early tumour stages (T1+T2) in comparison with late tumour stages (T3+T4) (Figure 3b). Regarding survival, patients with low *SIRT6* expression presented better disease-free survival than patients with high expression (Figure S2).

#### 2.2.2. Histone Demethylases

Histone demethylases (HDM) remove methyl groups from arginine and lysine residues of target proteins. Histone demethylation can induce both gene activation and transcriptional silencing, depending on the protein complexes formed [135,136]. In oesophageal cancer, conflicting data have been reported for *KDM1A/LSD1* (lysine-specific histone demethylase 1) expression. Some authors reported significantly higher mRNA and protein expression in ESCC compared with the corresponding normal oesophagus and precancerous tissues [122,123,124], whereas Chen and collaborators observed decreased *KDM1A* expression in tumour tissue [48]. These discrepancies in expression might be due to the use of different detection methods, as well as with the number of samples tested and the tissue status (fresh-frozen *versus* formalin-fixed). KDM1A expression also directly correlated with tumour size, lymphatic and vascular invasion, nodal and distant metastases and advanced tumour stage [48,122,124,125]. An association was observed between low KDM1A expression and better overall and disease-free survival [122,124,125]. Furthermore, KDM5B was significantly more expressed in the cytoplasm of ESCC cells compared to normal mucosa [126] and its nuclear expression was associated with histologic grade, as poorly differentiated tumours displayed significantly lower nuclear KDM5B levels [126]. A significant correlation was found between high KDM6A/UTX (ubiquitously transcribed X chromosome tetratricopeptide repeat protein) and better overall and disease-free survival in ESCC and it remained an independent prognostic factor in multivariable analysis [127]. In addition, frequent truncating mutations were observed in the *KDM6A* gene [43]. The expression of KDM7C/PHF2 (PHD finger protein 2) was higher in ESCC compared with normal tissue [126].

Most studies regarding HDM expression report up-regulation in oesophageal cancer compared to normal mucosa, suggesting an oncogenic role for these proteins (Table 2). In the TCGA dataset, we found that ESCC displayed significantly higher *KDM1A*, *KDM5B* and *KDM7C* levels than EAC (Figure 2h and Figure S1); conversely, EAC showed *KDM4C* and *KDM6A* up-regulation in comparison with ESCC (Figure S1). Furthermore, early tumour stages (T1+T2) display higher levels of *KDM6A* than late stages (T3+T4), as depicted in Figure 3c.

#### 2.2.3. Deubiquitinating Enzymes

Deubiquitinating enzymes mediate the removal of ubiquitin from substrate proteins. Similar to previous protein families, deubiquitination is able to promote transcription activation, as well as gene silencing in a context-dependent manner [99,137]. In ESCC, *USP14* (ubiquitin specific peptidase 14) levels were significantly increased compared to the corresponding normal tissue [129,130]. Furthermore, high USP14 levels positively correlated with distant metastasis and poor overall survival, constituting an independent prognostic factor for ESCC [130]. PSMD14 (proteasome 26S subunit, non-ATPase 14) was also overexpressed in ESCC in comparison with adjacent oesophagus [131]. Additionally, using the OncoLnc database [138] these authors observed poor overall survival for patients with high level of PSMD14 [131]. Moreover, *BAP1* (BRCA1 associated protein 1) was found to harbour inactivating mutations [43].

Altogether, deubiquitinating enzymes contribute to oesophageal cancer aggressiveness (Table 2). In the TCGA dataset, ESCC displayed significantly higher expression levels of *PSMD14* than EAC (Figure 2i).

### 2.3. DNA Writers

#### DNA Methyltransferases

DNA methyltransferases (DNMT) add methyl groups to DNA. Although DNA methylation is commonly associated with gene repression, DNMT has been also implicated of transcriptional activation [139]. In oesophageal carcinomas, *DNMT1* was significantly more expressed and in a larger percentage of cells than in normal oesophageal tissue [78,79,80,81]. In contrast, one study reported loss of DNMT1 expression in EAC and ESCC in comparison with normal epithelium, but this difference has not been quantified [108]. DNMT1 expression positively associated with lymph node invasion and relapse; DNMT1 positive patients presented better prognosis [79,82], and, in multivariable analysis, DNMT1 expression was an independent prognostic factor. DNMT1 expression was also directly associated with global methylation levels in ESCC [82]. *DNMT3B* was up-regulated in cancer samples compared with paired normal mucosa both at the mRNA and protein level and positively correlated with distant metastasis, poor response to curative-intent treatment and lower pathologic response [84,85,86]. Patients with DNMT3B expression had worse overall and disease-free survival and, in multivariable analysis, DNMT3B positivity was an independent prognostic factor [86]. MGMT (O-6-methylguanine-DNA methyltransferase) expression seems to be progressively lost from normal oesophageal tissue, basal cell hyperplasia, dysplasia to ESCC (although these differences have not been quantified) [140]. *MGMT* down-regulation in oesophageal cancer compared with the corresponding normal mucosa has also been reported [87,88]. Interestingly, *MGMT* transcriptional inactivation and loss of protein expression, in ESCC, were significantly associated with promoter hypermethylation [87,88,140]. Accordingly, *MGMT* methylation frequency in neoplastic tissue was higher than in the normal mucosa [87,141]. *MGMT* methylation was more frequent in poorly or moderately differentiated oesophageal tumours associated with depth of invasion, nodal status, the existence of metastasis and clinical stage [85,87,142]. Among cancer patients who received chemotherapy with alkylating agents, those displaying *MGMT* methylation had better prognosis than those without *MGMT* methylation [87].

Overall, alterations in DNMT proteins expression seem to favour oesophageal cancer progression (Table 1). Concerning differences between oesophageal subtypes, ESCC presented significantly higher *DNMT1*, *DNMT3B* and *MGMT* expression levels than EAC in the TCGA dataset (Figure 2j and Figure S1). Also, *DNMT1* was less expressed in cases with lymph node invasion in comparison with samples without lymph node metastisation (Figure 3d).

### 2.4. DNA Erasers

#### DNA Demethylases

DNA demethylases (TET) are critical enzymes of the pathway responsible for the removal of methyl groups from DNA. Unmethylated DNA is generally associated with gene transcription, although TET1 also participates in gene silencing [143]. *TET2* (ten-eleven translocation 2) expression was lower in ESCC than in normal mucosa and its expression in tumour tissue correlated with lymph node invasion [133,134]. In contrast, data regarding *TET3* expression are conflicting, with some authors showing down-regulation [134] and others up-regulation [133] in ESCC comparatively to non-tumour tissue. In both studies the same methodology was used to evaluate *TET3* expression, although Murata and colleagues [133] assessed a larger cohort, with almost twice the number of samples than that of Shi and collaborators [134]. In silico analysis of the TCGA data revealed a non-significant association for *TET3* overexpression in oesophageal tumour tissue in comparison with normal mucosa (Figure S3).

The available data on TET proteins allows us to conclude that they also associate with a malignant phenotype in oesophageal cancer (Table 2). We have further observed that *TET2* and *TET3* were significantly more expressed in ESCC than in EAC (Figure 2k and Figure S1).

### 2.5. Chromatin-Remodelling Enzymes

These enzymes induce chromatin alterations by affecting the interaction between histones and DNA in the nucleosome and through the modification of histones. Accordingly, their activity can induce gene expression or transcriptional repression [144,145]. Luo and collaborators [146] evaluated *YY1* (yin and yang 1) expression in ESCC and found it higher in tumours than in adjacent oesophagus and normal epithelium from healthy donors. YY1 protein was up-regulated in ESCC with lymph node invasion and its expression was more intense in late-stage tumours (III/IV) in comparison with early stage tumours (I/II) [147]. Expression of ARID1A (AT-rich interactive domain 1A), a member of the switch/sucrose non-fermentable (SWI/SNF) chromatin-remodelling complex, positively associated with the infiltrative growth pattern in ESCC [148]. In a study using feature selection algorithms and decision tree models, RUVBL1 (RuvB Like AAA ATPase 1) was proposed as a useful biomarker to discriminate normal oesophageal tissue from ESCC, especially in combination with CNIH (protein cornichon homolog) [149]. *BAZ1A* (bromodomain adjacent to zinc finger domain 1A) was found to be amplified and overexpressed in oesophageal cancer tissues and cell lines [150].

Remarkably, chromatin-remodelling enzymes also potentiate oesophageal cancer development. Using TCGA data, we have observed that *YY1* was more frequently expressed in ESCC in comparison with EAC (Figure 2l).

## 3. *In Vitro* Studies in Oesophageal Cancer

This section reviews *in vitro* data from the genes for which information was available for comparisons between oesophageal cancer *versus* normal tissue expression, although this is not an exhaustive account of all the cellular effects induced by the enzymes that mediate epigenetic modifications. These reports are based on experiments using oesophageal cancer cell lines, focusing on functional studies addressing several parameters of tumour biology, such as cell proliferation, apoptosis, motility, and invasion. Additionally, *in vivo* studies were included when available.

### 3.1. HDACs/HATs

#### 3.1.1. Histone Deacetylases

A considerable number of studies is available in the literature addressing the role of HDAC proteins in oesophageal cancer. The results regarding HDAC1 expression in cell lines are discrepant: some studies report down-regulation in oesophageal cancer cell lines compared with a benign epithelial cell line [108], whereas others point to increased expression in neoplastic cells [118]. It should be noted that these studies evaluated different cell lines and some degree of heterogeneity is expected among distinct cellular models. *HDAC1* knockdown caused reduction of cell growth, migration and invasion capacities of ESCC cells [105,118]. The levels of apoptosis and DNA damage in cells with silenced *HDAC1* increased after being exposed to ionising radiation [105]. Concerning HDAC2, there was significantly decreased expression in EAC and ESCC cell lines comparatively to a non-neoplastic cell line [108]. *HDAC2* silencing induced a decrease in ESCC cells invasiveness, accompanied by a reduction in metalloproteases expression [106]. *HDAC4* mRNA was overexpressed in oesophageal carcinoma cell lines and its down-regulation inhibited proliferation and migration, promoting cell cycle arrest [109]. *HDAC6* silencing led to a decrease in the proliferative, migratory and invasive capacities of ESCC cells, followed by cell cycle arrest at the G0/G1 phase [119,120]. The enzymatic activity of HDAC proteins was augmented in oesophageal cancer cell lines compared to a non-neoplastic cell line [108]. *SIRT1* mRNA and protein levels were up-regulated in most ESCC cell lines comparatively to a benign oesophageal epithelial cell line [110]. *SIRT3* silencing inhibited proliferation and induced apoptosis in oesophageal cancer cells [121], whereas *SIRT4* silencing augmented proliferation and migration of ESCC cells [116]. *In vitro* studies showed that *SIRT6* overexpression induced cell growth, autophagy and increased the proportion of cells in the G2/M phase [117].

Overall, and except for *SIRT4*, HDACs seem to play an oncogenic role in oesophageal cancer (Table 1). This result also corroborates the findings of tissue-based studies, in which most of these genes were found to be overexpressed in oesophageal cancer compared to normal mucosa.

#### 3.1.2. Histone Acetyltransferases

Concerning histone acetyltransferases, *KAT1* knockdown reduced the viability of oesophageal cancer cell lines and induced cell cycle arrest [39]. In another study, *KAT13B* silencing reduced ESCC cells proliferation, viability, migration and invasion, arrested cell cycle and inhibited tumour growth *in vivo* [47].

Although to a lesser extent, with only two genes assessed, the results for histone acetyltransferases resemble those obtained for HDACs, with *KAT1B* and *KAT13B* promoting the development of oesophageal cancer, both in cell lines and animal studies, and being up-regulated in oesophageal cancer tissues (Table 1).

### 3.2. HMTs/HDMTs

#### 3.2.1. Histone Methyltransferases

Concerning histone methyltransferase enzymes, *KMT3C*/*SMYD2* mRNA and protein were overexpressed in some oesophageal cancer cell lines compared to normal oesophagus [50]. Upon *KMT3C* silencing, there was diminished proliferative ability and cell cycle arrest at the G0/G1 phase [50], whereas *KMT3E* silencing induced a decrease in proliferation, migration and invasion of oesophageal cancer cell lines and inhibition of tumour invasion *in vivo*, as well as up-regulation of *KMT8* mRNA and protein levels [51,52]. Furthermore, *KMT5A*/*SET8* knockdown suppressed the proliferative, migratory and invasive capacities of cancer cells *in vitro*, as well as tumour development *in vivo* and promoted apoptosis of oesophageal cancer cells [53].

The members of this family of proteins also seem to work as oncogenes in functional assays, lending support to the findings from expression studies, where HMT proteins were overexpressed in oesophageal cancer in comparison with normal tissue (Table 1).

#### 3.2.2. Histone Demethylases

*In vitro* experiments with oesophageal cancer cell lines focusing on the study of HDM have shown a decrease in the migration and invasion capabilities of cancer cells upon *KDM1A* silencing [122,124]. *KDM1A* knockdown also suppressed the glycolytic pathway and promoted mitochondrial respiration [124]. Moreover, *KDM4C*/*GASC1* (gene amplified in squamous cell carcinoma 1) was amplified and overexpressed in ESCC cell lines [151], whereas KDM5B/JARID1B (Jumonji at rich interactive domain 1B) was up-regulated in ESCC cell lines compared with normal oesophageal cells [152]. Upon *KDM6A* silencing an increase in the proliferating ability of oesophageal cancer cells, as well as the induction of epithelial-to-mesenchymal transition (EMT) was observed [127]. Furthermore, *KDM7B*/*PHF8* (PHD finger protein 8) down-regulation inhibited anchorage-dependent and -independent growth, as well as the proliferative, migratory and invasive abilities of ESCC cells and induced apoptosis [128]. Using nude mice, those authors observed decreased tumorigenicity of oesophageal cancer cells and confirmed both the inhibition of proliferation and promotion of apoptosis upon *KDM7B* knockdown [128].

The results concerning HDM enzymes are more difficult to interpret, since the genes for which we found studies on tissue expression do not entirely match with the ones investigated in *in vitro* functional assays. However, in the first set of results, HDM genes (*KDM1A, KDM5B, KDM6A* and *KDM7C*) were mostly overexpressed in tumour tissue comparatively to normal oesophageal epithelium. Accordingly, genes evaluated in the latter group of studies (*KDM1A, KDM6A* and *KDM7B*) suggest an oncogenic role for HDM, although *KDM6A* seems to be a tumour-suppressor gene (Table 2).

#### 3.2.3. Kinases

Concerning the kinase family, *AURKA* overexpression promoted migration and invasion of oesophageal cancer cell lines and increased tumour size *in vivo* [58,61]. Conversely, *AURKA* silencing inhibited migration, colony formation and cell growth and increased the expression of apoptotic markers [57,59]. Furthermore, the size of tumour xenografts decreased upon *AURKA* knockdown [59]. *AURKB* silencing inhibited anchorage-independent growth, colony formation and cell growth and increased the expression of apoptotic markers [59,62]. Moreover, the size of tumour xenografts decreased upon *AURKB* knockdown [59,62]. Survivin levels increased in oesophageal cancer cells in comparison with a human epithelial cell line [153]. *BIRC5* knockdown decreased cell proliferation and induced apoptosis in oesophageal cancer cells [154], whereas *PRKCI* silencing decreased proliferation, migration, invasion and growth of anchorage-independent colonies of oesophageal cancer cells and induced apoptosis [64,65]. In mice, *PRKCI* depletion gave rise to smaller tumours and less lung metastases than in the control group [65].

The kinases evaluated in functional studies behaved as oncogenes, promoting oesophageal cancer development (Table 1). This result corroborates the findings concerning expression studies in primary cancer tissues, in which kinases were up-regulated.

#### 3.2.4. Ubiquitin-Activating/-Conjugating/-Ligation Enzymes

Concerning ubiquitin-related enzymes, *BMI1* down-regulation was shown to suppress cell viability and increase radiosensitivity of ESCC cells after irradiation, as well as to inhibit tumour formation in nude mice [69]. Using *in vitro* and *in vivo* models, increased apoptosis, decreased proliferation and tumorigenicity of ESCC cells was observed upon *FBXO31* silencing. *FBXO31* knockdown was also shown to sensitise ESCC cells to cisplatin therapy [70]. Upon *UBE2C* silencing there was a decrease in cell proliferation of EAC and ESCC cell lines [73], whereas *RNF113A* knockdown resulted in reduced proliferation and increased apoptosis, as well as inhibition of the migratory and invasive capacities of ESCC cell lines and decreased tumour growth in nude mice [77].

In *in vitro* studies, the members of this family of enzymes play an oncogenic role (Table 1), a result that supports the tissue-based experiments, with ubiquitin-related proteins being more expressed in carcinomas than in the normal counterpart.

#### 3.2.5. Deubiquitinating Enzymes

*In vitro* assays demonstrated that *USP14* knockdown decreased proliferation, migration, and invasion of ESCC cells and also reduced tumorigenicity in mice. Furthermore, *USP14* silencing inhibited EMT in oesophageal cancer cell lines, causing increased expression of E-cadherin and down-regulation of vimentin and N-cadherin [129]. Likewise, *PSMD14* knockdown blocked EMT in ESCC cells, through decreased N-cadherin and vimentin levels and augmented E-cadherin levels [131].

These data strengthen the notion that deubiquitinating enzymes behave as oncogenes in oesophageal cancer, promoting aggressive features in tumour cells (Table 2). This is in accordance with the tissue data, which demonstrated an up-regulation of deubiquitinating enzymes in cancer samples in comparison with normal epithelium.

### 3.3. DNMT/TETs

#### 3.3.1. DNA Methyltransferases

Functional studies addressing DNMTs demonstrated that *DNMT1* knockdown reduced proliferation, viability, invasion and metastasis in ESCC cells and induced apoptosis [79,83]. Furthermore, *DNMT1* silencing inhibited tumour growth in a mouse model [83]. *DNMT3B* down-regulation induced apoptosis and cell cycle arrest, increasing autophagy and sensitising cells to irradiation and treatment with cisplatin [86]. Tumour growth *in vivo* was also significantly suppressed upon *DNMT3B* knockdown, as well as the invasive ability of oesophageal cancer cells [86].

Similar to other families of enzymes presented thus far, DNMT proteins also exert an oncogenic role in functional studies (Table 1). This result corroborates the findings from tissue-based expression experiments, in which DNMTs were up-regulated in oesophageal tumour tissue comparatively to normal epithelium.

#### 3.3.2. DNA Demethylases

As far as we know, only one study evaluated the role of TET using functional assays in oesophageal cancer. Accordingly, it demonstrated that ectopic *TET1* expression suppressed colony formation and augmented apoptosis in ESCC cells [132].

As mentioned for HDM, the results from TET are difficult to interpret, since we could only find tissue expression data regarding *TET2* and *TET3* [133,134], whereas just one in vitro study addressed the role of *TET1* [132]. *TET2* was up-regulated in oesophageal cancer in comparison with normal epithelium, suggesting an oncogenic role for this gene and, conversely, *TET1* seems to behave as a tumour-suppressor gene. Data are conflicting regarding TET3 expression, with studies reporting both over- and underexpression in tumour tissue compared with normal mucosa (Table 2).

### 3.4. Chromatin-Remodelling Enzymes

Finally, regarding chromatin-remodelling enzymes, *in vitro* studies demonstrated that *YY1* overexpression suppressed ESCC growth, whereas, surprisingly, also promoted invasiveness of oesophageal cancer cells. The authors speculate that YY1 is involved in ESCC proliferation inhibition but fosters metastisation [146,147]. Interestingly, *YY1* up-regulation endowed radioresistance to ESCC cells [146].

*YY1* results are conflicting, since this gene seems to act simultaneously as an oncogene and a tumour-suppressor in functional assays. In contrast, the enzyme is overexpressed in tumour tissue compared to normal oesophageal mucosa, supporting an oncogenic role.

## 4. Conclusions

Characterisation of the epigenetic landscape of oesophageal cancer is an ongoing work. Although many studies focused on specific epigenetic mechanisms and players, a comprehensive understanding of this disease at the epigenetic level is yet to be achieved. Considering the lack of targeted therapies for this specific tumour type, it is vital that new strategies are developed to tackle this malignancy and decrease the high mortality rates. Additionally, biomarkers for screening are also in need because currently used methods are invasive and, to some degree, ineffective. Epigenetic players have been arising as potential solutions for these problems. In this review, we have provided an overview of studies addressing the expression of enzymes engaged in DNA and/or histone modifications at the mRNA and protein level, emphasising differences between normal and malignant oesophageal tissue. Furthermore, we correlated the expression of those enzymes with standard clinicopathological parameters, such as tumour grade, stage, and lymph node metastasis, and compared their expression among the two major histological subtypes. The results collected herein, as well as the analysis that we performed based on the TCGA dataset, demonstrate that epigenetic players may constitute attractive therapeutic targets in oesophageal cancer, since they are mostly overexpressed in tumour cells in comparison with normal oesophageal mucosa. A possible explanation for the concomitant up-regulation of writers and erasers in oesophageal cancer lies in the requirement of tumour cells’ plasticity, needed for the tissues’ adaptation to the environmental conditions. Thus, having high levels of enzymes responsible for DNA and histone modifications may allow a rapid swift of the epigenome, increasing the survival chances of cancer cells. The amount of these enzymes is also context-dependent, with expression levels changing with tumour stage (as previously referred for KMT1A and HDAC1, among others). Furthermore, the availability of enzyme-specific targets is a limitation factor, since the lack of targets for erasers will determine that writers will prevail and vice-versa. Among histological subtypes, most of the genes studied are more expressed in ESCC than in EAC. Moreover, *in vitro* studies suggest that almost all deregulated genes implicated in oesophageal cancer addressed in this review act as oncogenes, fostering neoplastic development and progression. Accordingly, we are tempted to speculate whether epigenetic drugs (HDAC and DNMT inhibitors) already in use for some haematologic malignancies [155,156] might be as effective in oesophageal cancer. The goal in this solid tumour would be to induce reprogramming of neoplastic cells, sensitising them to routinely used cytotoxic drugs [34]. However, the therapeutic use of epigenetic drugs in oesophageal cancer is a relatively recent research field, with only a few clinical studies already completed and others still ongoing. A phase I clinical trial found significant clinical and epigenetic responses in patients harbouring resectable oesophageal and gastric adenocarcinoma following treatment with 5-azacitidine, a DNA demethylating agent, prior to neoadjuvant chemotherapy, suggesting that this drug could augment tumour sensitivity to conventional therapy [157]. Other clinical trials have been completed, but the results have not yet been published. Further studies using large patient cohorts are required to validate the already sizeable preclinical data and test the efficacy of epigenetic drugs against oesophageal cancer. The next couple of years will surely result in progress in this exciting area of research and hopefully also pave the way for improved therapeutic strategies that may alleviate oesophageal cancer burden.

## Figures and Tables

**Table 1:** Summary of the most relevant publications concerning the role of DNA/histone writers involved in oesophageal cancer.

Protein Family	Gene	Tumour Expression (in Comparison with Normal Mucosa)	Survival	Functional Assays	References
**HAT**	*KAT1*	Overexpressed	NA	Oncogene	[39]
	*KAT3B*	Overexpressed	High: Poor OS and DFS	NA	[40,41]
	*KAT13B*	Overexpressed	High: Poor OS, DSS and PFS	Oncogene	[44,45,47]
**HMT**	*KMT1A*	Overexpressed	NA	NA	[48]
	*KMT1D*	Overexpressed	High: Poor OS	NA	[49]
	*KMT3C*	NA	High: Poor OS	Oncogene	[50]
	*KMT3E*	Overexpressed	High: Poor OS	Oncogene	[51,52]
	*KMT5A*	NA	High: Poor OS	Oncogene	[53]
	*KMT6A*	Overexpressed	Positive/Aberrant: Poor OS, DFS, DSS and PFS	NA	[48,54,55,56]
**Kinases**	*AURKA*	Overexpressed	Positive: Poor OS and DFS	Oncogene	[57,58,59,60,61]
	*AURKB*	Overexpressed	High: Poor OS	Oncogene	[59,62]
	*PKC*	Overexpressed	NA	NA	[63]
	*PRKCI*	Overexpressed	High: Poor OS	Oncogene	[64,65]
	*PAK1*	Overexpressed	High: Poor OS	NA	[66]
	*PAK2*	Overexpressed	NA	NA	[67]
**Ubiquitin -related enzymes**	*SMURF2*	NA	Positive: Poor OS	NA	[68]
	*BMI1*	Overexpressed	High: Poor OS	Oncogene	[54,69]
	*FBXO31*	Overexpressed	High: Poor OS	Oncogene	[70,71]
	*FBXW7*	NA	High: Good OS	NA	[72]
	*UBE2C*	Overexpressed	High: Poor RFS	Oncogene	[73,74,75,76]
	*RNF113A*	Overexpressed	High: Poor OS	Oncogene	[77]
**DNMT**	*DNMT1*	Overexpressed	NA	Oncogene	[78,79,80,81,82,83]
	*DNMT3B*	Overexpressed	Positive: Poor OS and DFS	Oncogene	[84,85,86]
	*MGMT*	Underexpressed	NA	NA	[87,88]

OS—overall survival, DFS—disease-free survival, DSS—disease-specific survival, PFS—progression-free survival, RFS—relapse-free survival, NA—not available.

**Table 2:** Summary of the most relevant publications concerning the role of DNA/histone erasers involved in oesophageal cancer.

ProteinFamily	Gene	Tumour Expression (in Comparison with Normal Mucosa)	Survival	Functional Assays	References
**HDAC**	*HDAC1*	Overexpressed	NA	Oncogene	[48,105,118]
	*HDAC2*	Overexpressed	NA	Oncogene	[48,78,106,107]
	*HDAC3*	Overexpressed	NA	NA	[93]
	*HDAC4*	Overexpressed	High: Poor OS and PFS	Oncogene	[109]
	*HDAC5*	Overexpressed	NA	NA	[48]
	*HDAC6*	NA	NA	Oncogene	[119,120]
	*SIRT1*	Overexpressed	High: Poor OS and DFS	NA	[48,110,111,112]
	*SIRT3*	Overexpressed	High: Poor OS and DFS	Oncogene	[113,114,115,121]
	*SIRT4*	NA	High: Good OS and DFS	Tumour-suppressor	[116]
	*SIRT6*	Overexpressed	NA	Oncogene	[117]
**HDM**	*KDM1A*	Overexpressed/Underexpressed	High: Poor OS and DFS	Oncogene	[48,122,123,124,125]
	*KDM5B*	Overexpressed	NA	NA	[126]
	*KDM6A*	Overexpressed	High: Better OS and DFS	Tumour-suppressor	[127]
	*KDM7B*	NA	NA	Oncogene	[128]
	*KDM7C*	Overexpressed	NA	NA	[126]
**Deubiquitinating enzymes**	*USP14*	Overexpressed	High: Poor OS	Oncogene	[129,130]
	*PSMD14*	Overexpressed	High: Poor OS	Oncogene	[131]
**TET**	*TET1*	NA	NA	Tumour-suppressor	[132]
	*TET2*	Underexpressed	NA	NA	[133,134]
	*TET3*	Overexpressed/Underexpressed	NA	NA	[133,134]

OS—overall survival, PFS—progression-free survival, DFS—disease-free survival, NA—not available.

## Supplementary Materials

The following are available online at https://www.mdpi.com/1422-0067/21/10/3522/s1.
