# Supplementary material for: Epigenetic Alterations in Oesophageal Cancer: Expression and Role of the Involved Enzymes
Source: Int J Mol Sci. 2020 May 15;21(10):3522. doi: 10.3390/ijms21103522 (PMC7278932; doi:10.3390/ijms21103522)
Supplement: Supplementary file 1 [file ijms-21-03522-s001.pdf]

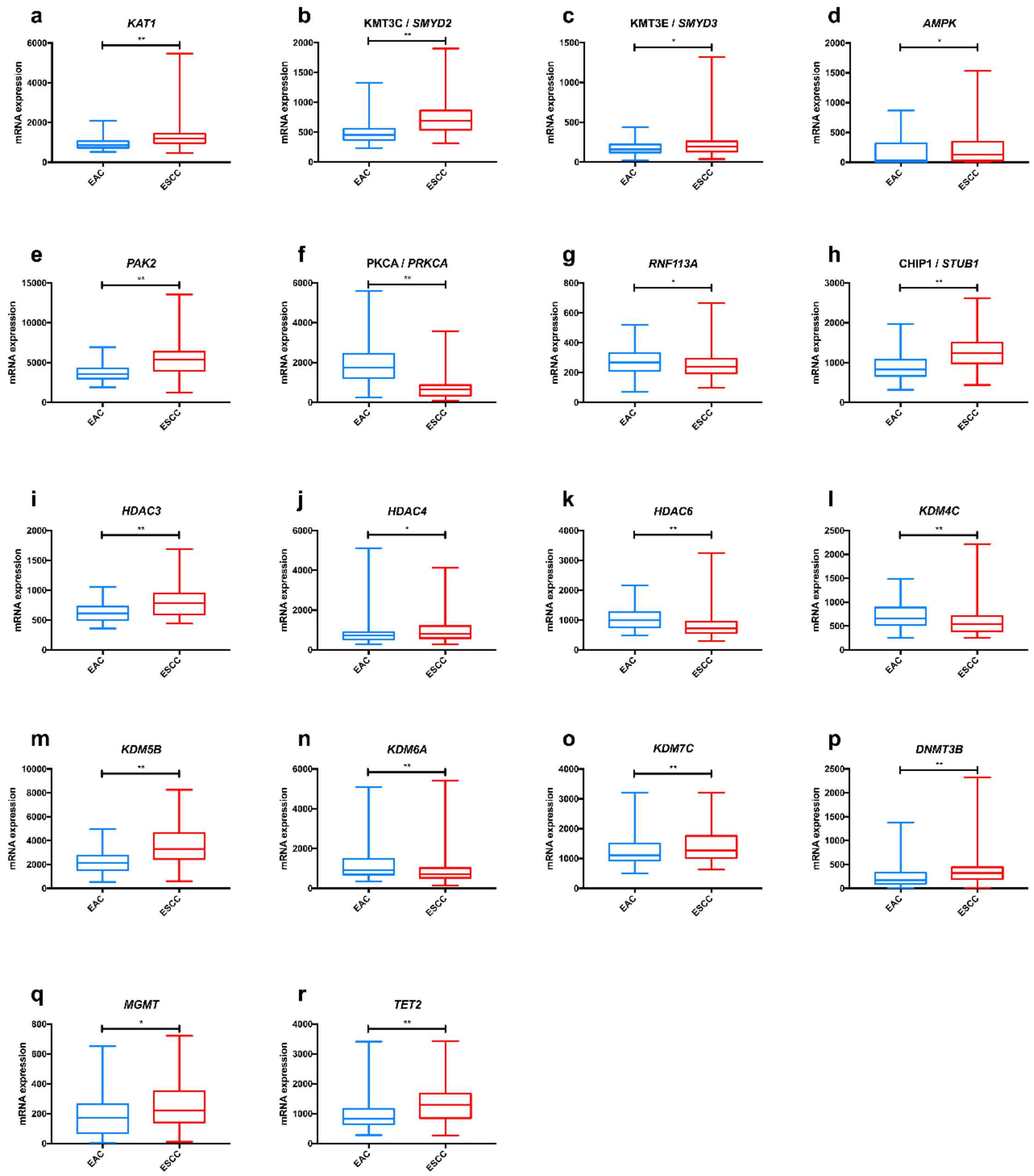


**Figure S1.** Examples of relevant modifications in the expression of enzymes involved in epigenetic alterations in oesophageal cancer, based on TCGA data analysed through the cBioPortal for Cancer Genomics resource (data extracted from cBioPortal and graphs built using GraphPad Prism 7). Differences in mRNA expression of *KAT1* (a), KMT3C (b), KMT3E (c), *AMPK* (d), *PAK2* (e), *PRKCA* (f), *RNF113A* (g), CHIP1 (h), *HDAC3* (i), *HDAC4* (j), *HDAC6* (k), *KDM4C* (l), *KDM5B* (m), *KDM6A* (n), *KDM7C* (o), *DNMT3B* (p), *MGMT* (q) and *TET2* (r) between EAC and ESCC; Abbreviations: EAC – oesophageal adenocarcinoma; ESCC – oesophageal squamous cell carcinoma; * $p < 0.05$, ** $p < 0.001$.

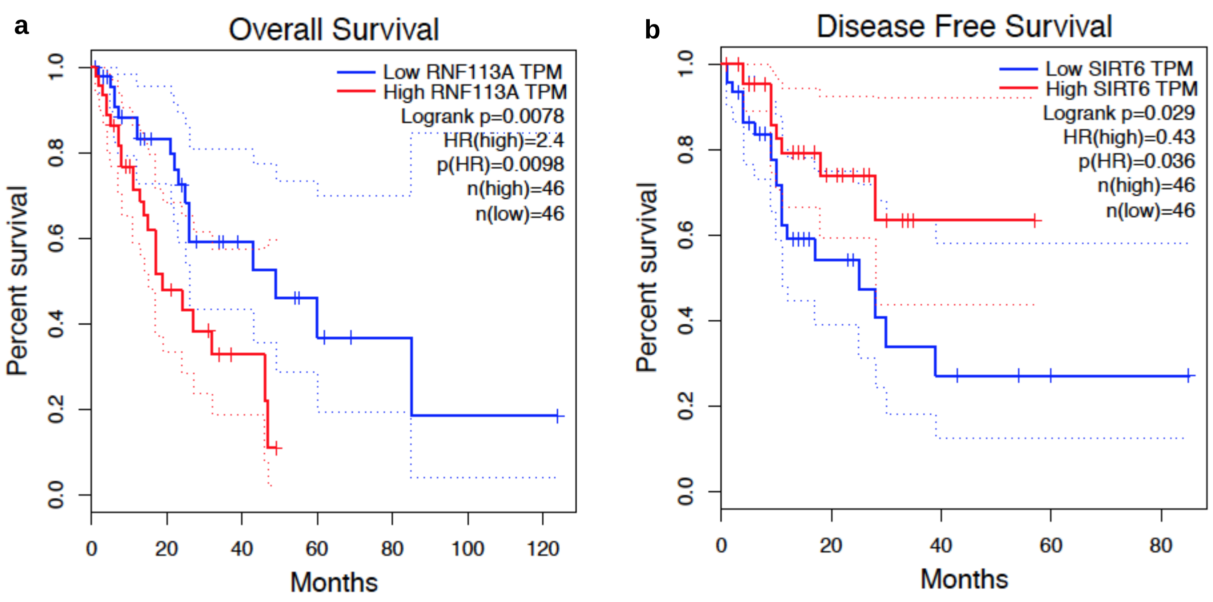


**Figure S2.** Kaplan–Meier curves showing survival analyses regarding the expression of enzymes involved in epigenetic alterations in oesophageal cancer, based on TCGA data analysed through the GEPIA resource (survival curves extracted from GEPIA). (a) Oesophageal cancer patients with high *RNF113A* expression display worse overall survival than those with low *RNF113A* expression; (b) Oesophageal cancer patients with low *SIRT6* expression display worse disease-free survival than those with high *SIRT6* expression.

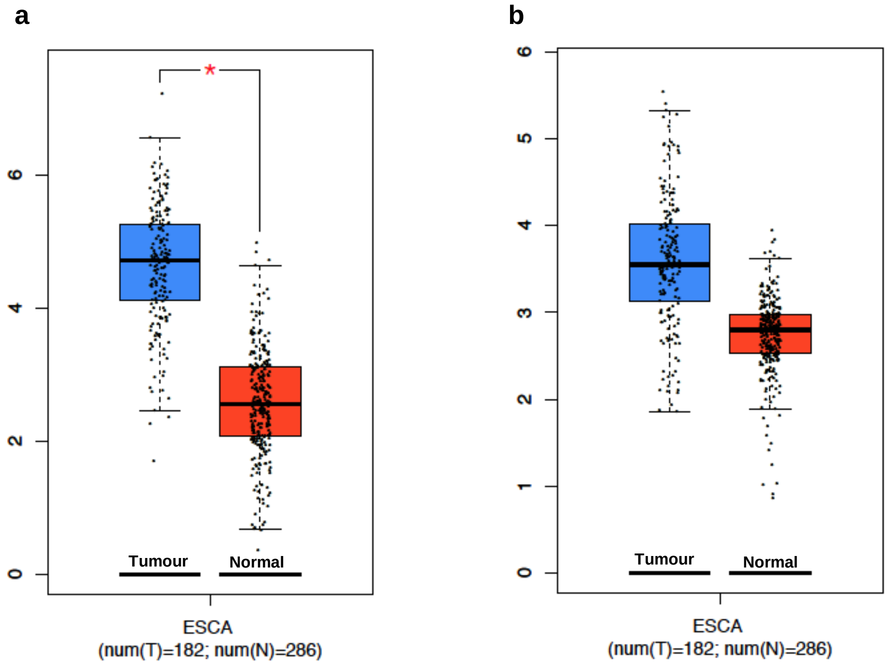


**Figure S3.** Boxplots showing differences in the expression of enzymes involved in epigenetic alterations in oesophageal cancer, based on TCGA and GTEx data analysed through the GEPIA resource (boxplots extracted from GEPIA). (a) Oesophageal cancer samples display higher *AURKA* expression levels than normal oesophagus samples; (b) Oesophageal cancer samples display higher *TET3* expression levels than normal oesophagus samples. Abbreviations: T – tumour, N – normal, num - number; * $p < 0.01$.
